# Association of Some Polymorphisms in the VDR Gene, CYP17 Gene and SRD5A2 Gene and Prostate Cancer among Lebanese Men

**DOI:** 10.22034/APJCP.2017.18.1.93

**Published:** 2017

**Authors:** Asmahan A El Ezzi, Vladyslav G Boyko, Monika T Baker, Wissam R Zaidan, Kalim M Hraiki, Mohammad A El-Saidi, Ruhul H Kuddus

**Affiliations:** 1 *Lebanese Atomic Energy Commission, Beirut, *; 2 *Department of Biochemistry, Lebanese University, Hadath,*; 5 *Department of Urology, Al-Salam Hospital, Tripoli, Lebanon,*; 3 *Department of Chemistry, *; 4 *Department of Biology, *; 6 *Department of Strategic Management and Operations, Utah Valley University, Orem, Utah, USA. *

**Keywords:** Prostate cancer (PCa), single nucleotide polymorphism (SNP), TA repeat polymorphism, VDR gene

## Abstract

**Aims:**

The goal of the study was to investigate possible association of some single nucleotide polymorphisms (SNPs) in the VDR gene (the FokI, BsmI, ApaI and TaqαI loci), and the CYP17 gene (the MspA1I locus), and 0 or 9 TA repeats in the SRD5A2 gene, and prostate cancer (PCa) among Lebanese men.

**Materials and Methods:**

Blood DNA of 69 subjects with confirmed PCa and 69 controls, all about 50 years of age or older, was subjected to PCR or PCR-restriction fragment-length polymorphism (PCR-RFLP) analyses, and the risk-bearing and the protective alleles were identified. The odds ratio (OR) of having a genotype and the relative risk (RR) of developing PCa were calculated. In addition, the distributions of homozygosis and heterozygosis in the risk-bearing alleles and the protective alleles among the control and the PCa groups were compared.

**Results:**

The f allele of the VDR FokI locus and the (TA) 9 repeat allele of the SRD5A2 gene were found to be associated with increased risks of PCa (p = 0.006 and 0.050, respectively). Homozygosis in the risk-bearing alleles was rare both in the control and the PCa groups. A higher fraction of the controls compared to the PCa group was double-homozygous in the two protective alleles (52.2% for controls, 24.6% for PCa group, p = <0.001).

**Conclusions:**

To the best of our knowledge, this is the first genetic study demonstrating the association of certain polymorphisms of the VDR gene and the SDR5A2 gene and increased risk of PCa among Lebanese men. Our study also indicates that the overall polymorphism profile of all genes involved in the prostate physiology is likely to be a better indicator for PCa risk than the polymorphisms in the individual genes.

## Introduction

Prostate cancer (PCa) is the second most commonly diagnosed cancer for males and the second leading cause of cancer deaths among males of all races (ACS, 2016; Seigel et al., 2011). The possible risk factors for PCa include age, diet, obesity, lifestyle, smoking, infections and inflammation of the prostate gland, vasectomy, exposure to certain types of chemicals, and genetic predisposition (ACS, 2016). The incidence of PCa is highest among Africans, followed by Caucasians and Mongolians (Tao et al., 2015; Hsing et al., 2000). Identification and validation of specific genetic markers could be helpful in the screening and early diagnosis of prostate cancer. 

Polymorphisms in several genes, including the vitamin D receptor (VDR) gene, the androgen receptor (AR) gene, the cytochrome P-450 17 alpha-hydroxylase/C(17, 20)-lyase (CYP17) gene, and the steroid 5 alpha-reductase type 2 (SRD5A2) gene, have been implicated in PCa development (Wang et al., 2016; Dianat et al., 2009, Ntais et al., 2003). The VDR gene encodes a ligand-inducible transcription factor that regulates many physiological processes including cell growth, embryonic development and metabolic homeostasis (Gene Cards, 2016; Feldman and Malloy, 2014). The CYP17 gene encodes an enzyme that controls a rate-limiting step in androgen biosynthesis (Gene Cards, 2016; Chen et al., 2014). SRD5A2 gene encodes an enzyme that converts testosterone to dihydrotestosterone (Gene Cards, 2016; Boer et al., 2016), the more biologically active form of the hormone. Since the prostate gland is an androgen-regulated organ, polymorphisms in the CYP17 and SRD5A2 genes could be associated with PCa.

A number of previous molecular epidemiological studies reported certain polymorphisms of these genes as protective or risk-bearing factors for PCa, but other studies found no such associations (Wang et al., 2016; Norman, 2006; Ntais et al., 2003). This may be due to racial and ethnic differences (CDC, 2016; Hsing et al., 2000), and the presence of both protective and risk-bearing polymorphisms in different genes in almost every individual (Kitts et al., 2014; Latil et al., 2001). In the present study, we examined a number of polymorphisms in the VDR gene, the CYP17 gene and the SRD5A2 gene among Lebanese men with PCa and healthy Lebanese men with no prostate disease. We analyzed the association of polymorphisms in certain alleles of each of the three genes individually as well as the overall polymorphisms of the genes as a risk factor for PCa.

## Materials and Methods

*Study population*

Men enrolled in the study were volunteers who participated in the prostate cancer campaigns organized by Dr. Asmahan A. El Ezzi at the Lebanese Atomic Energy Commission in collaboration with many hospitals and medical centers in Lebanon. All subjects were Caucasian Lebanese men between about 50 and 70 years of age. Informed consent to participate in the study for PSA screening, donation of blood for DNA extraction and storage, and using the DNA samples for molecular research was obtained from each of the subjects in accordance with the Declaration of Helsinki and following the guidelines of the Institutional Review Board of Lebanese University, Beirut, Lebanon. Prostate condition of the subjects was examined by measuring the serum prostate-specific antigen (PSA) level of all participants and, if consented, by digital rectal examination (DRE). Blood samples were withdrawn in non-fasting state, serum was separated and stored at -30^o^C till the day of PSA assay. Total PSA (PSA-T) assays were performed following the guidelines of the kits purchased from Immunotech (Marseille, France). For subjects with PSA-T level in the gray zone (i.e. between 4-10 ng/ml), a free PSA test (PSA-F) was also conducted, and the F/T PSA level was determined, in order to help differentiate benign prostate hyperplasia (BPH) and PCa. In addition, the International Prostate Symptom Score (IPSS) value was determined, and a trans-rectal prostate ultrasonography was conducted if needed. Histological analyses of prostate biopsies confirmed the diagnosis of prostate cancer. Sixty-nine subjects with confirmed PCa and 69 controls were included in the present study. The control subjects were volunteers who had normal PSA level, normal IPSS score, and normal DRE for two consecutive years. 

*DNA extraction and analysis*

Blood DNA was extracted using QiaAmp DNA Blood Mini Kit (Qiagen, Milan, Italy), and stored at -82^o^C before transporting to the United Sates in a temperature-controlled package for molecular analyses. DNA was quantified using a NanoDrop spectrophotometer (Thermo Fisher, Waltham, MA). The DNA samples with A_260_/A_280_ ratio between 1.8-2.0 were used for PCR-amplification of the appropriate DNA fragments for further analyses. The following primers were used: VDR Fok1- 5’TGCAGCCTTCACAGGTCATA3’ and 5’GGCCTGCTTGCTGTTCTTAC3’, VDR BsmI- 5’CAGTTCACGCAAGAGCAGAG3’ and 5’ACCTGAAGGGAGACGTAGCA3’; VDR ApaI- 5’ACGTCTGCAGTGTGTTGGAC3’ and 5’TCACCGGTCAGCAGTCATAG3’ (Falleti et al., 2010); VDR TaqαI- 5’CAGAGCATGGACAGGGAGCAA3’ and 5’GCAACTCCTCATGGCTGAGGTCTC3’ (Taylor et al., 1996); CYP17- 5’CATTCGCACCTCTGGAGTC3’ and 5’GGCTCTTGGGGTACTTG3’ (Feigelson et al., 1997); and SRD5A2- 5’GCTGATGAAAACTGTCAAGCTGCTGA3’ and 5’GCCAGCTGGCAGAACGCCAGGAGAC3’ (Forrest et al., 2005). The reaction mixture for PCR (15 µl) contained 1x master mix reaction buffer containing Taq DNA polymerase (Qiagen, Germantown MD), 20 pico moles of the two primers, and 20 ng of template DNA. The thermocycler (GeneAmp PCR 9700, ABI, Foster City, CA) was programmed as the following: 94^o^C for 5 minutes (one cycle), 94^o^C for 45 sec, 57-64^o^C (depending on the primer pairs used) for 45 sec, and 72^o^C for 60 sec, (35 cycles); 72^o^C for 5 minutes (one cycle), and soak at 4^o^C.

For the analysis of PCR-amplified DNA with various numbers of TA repeats of the SRD5A2 gene, the amplified DNA was directly resolved in 6.0% polyacrylamide gels. RFLP of various loci of the other genes was conducted by treating the PCR-amplified DNA with 2 units of an appropriate restriction endonuclease (i.e. ApaI, BsmI, FokI, or TaqαI for VDR gene and MspA1I for CYP17 gene; all from New England Biolab, Beverly, MA). The reaction mixture was incubated overnight in the reaction buffer and in the incubation-temperature as suggested by the manufacturer. The treated DNA was then resolved in a 6% polyacrylamide gel. The gels were stained with ethidium bromide and then documented using a digital camera. RFLP alleles are codominant but for convenience, the alleles having the repeat DNA or the restriction site were considered recessive. The band pattern for different genotypes are as the following: VDR FokI: FF- 157 bp, Ff- 157 bp, 121 bp and 36 bp, ff- 121 bp and 36 bp, VDR BsmI: BB- 236 bp, Bb- 236 bp, 197 bp and 39 bp, bb- 197 bp and 39 bp; VDR ApaI: AA- 211 bp, Aa- 211 bp, 172 bp and 39 bp, aa- 172 bp and 39 bp; VDR TaqαI: TT- 495 bp and 245 bp; Tt- 495 bp, 290 bp, 245 bp, and 205 bp, tt- 290 bp, 245 bp, and 205 bp; CYP17: A1/A1 (i.e. MM)- 459 bp, A1/A2 (i.e. Mm)- 459 bp, 335 bp and 124 bp, A2/A2 (i.e. mm)- 335 bp and 124 bp; and SRD5A2: 0/0- 98bp, 0/9- 98 bp and 116 bp, and 9/9- 116 bp. Both 0/9 and 9/9 genotypes of the SRD5A2 gene occasionally produced three additional large-sized artifact bands that were not scored. 

*Statistical analyses*

Whether the distribution of the genotypes and the frequency of alleles are in agreement with the Hardy-Weinberg equilibrium was tested by χ^2^ statistics at the 0.05 % level of significance. The null hypothesis that the population is in Hardy-Weinberg equilibrium was rejected if the χ^2^ value was >3.84. Association between PCa and the genotypes was assessed by calculating the odds ratio (OR) and the 95% confidence interval (CI). The OR was calculated using the formula proposed by Bland and Altman (2000), and the relative risk (RR) was calculated using the formula proposed by Sheshkin (2004). The OR, RR, 95% CI and p-values were calculated using MedCalc software (MedCalc, Mariakerke, Belgium). A value of RR >1 for a genotype is considered implicative of disease risk relative to the alternative genotype. The genotypic ratio among the controls and the subjects with PCa were compared using the two-tailed z test for the difference between two independent proportions at the 0.05 level of significance, where the null hypothesis H0 was: there is no significant difference between the two proportions, and the alternative hypothesis H1 was: there is a significant difference between the two proportions. The null hypothesis was rejected and the alternative hypothesis that there is a significant difference between the two tested proportions was supported only if the p-value was ≤ 0.05. The mean age of the control and PCa groups was compared using MedCalc Software. 

## Results

*Distribution of the genotypes and allele frequencies*

This study included 69 cases of PCa (mean age 66.4±8.5 years) and 69 male cohort controls (mean age 55.8±11.0 years). The mean age of the two groups is significantly different (p=<0.001). The distribution of homozygous dominant, homozygous recessive, and heterozygote genotypes are shown in Table 1. The distribution of the genotypes of almost all of the loci were in Hardy-Weinberg equilibrium except the ApaI locus of the VDR gene (χ^2^ = 9.88; p-value = 0.002), and the (TA)0-9 repeats of the SRD5A2 gene for the control group (χ^2^ = 5.02; p-value = 0.025). In these two loci, the ratio of the homozygous dominant, homozygous recessive, and heterozygous genotypes significantly deviated from the expected ratios.

The allele frequency for each of the examined loci of the three genes among the controls and the subjects with PCa is shown in Table 2. There is no significant difference in the allele frequencies of the VDR BsmI, ApaI and TaqαI loci, and the CYP17 MspA1I locus among the controls and the subjects with PCa. The f allele of the FokI locus of the VDR gene and the (TA) 9 allele of the SRD5A2 gene are overrepresented among the subjects with PCa compared to the controls (p-values 0.006 and 0.050, respectively).

*Genotype frequencies and genotypic ratios*

The ratio of the homozygote recessive or dominant genotypes and heterozygote genotypes for each of the alleles among the subjects with PCa and the controls are shown in Table 3. The OR of having a particular genotype for the subjects with PCa compared to the controls and the RR of PCa for having the genotype ratio are also shown in Table 3. In most cases, the ratio for the subjects with PCa and the controls is not significantly different except the following:

*VDR FokI*

The ratio of FF/Ff (and FF/ff) are lower for the subjects with PCa compared to the controls and the difference is statistically significant (OR: 0.43, 95% CI: 0.21-0.86, p-value = 0.017, and the corresponding RR: 0.68, 95% CI: 0.49-0.94, p-value =0.021). Also, the ratio of (Ff+ff)/FF is higher for the subjects with PCa compared to the controls and the difference is statistically significant (OR: 2.59, 95% CI: 1.29-5.15, p-value = 0.007, and the corresponding RR: 1.67, 95% CI: 1.14-2.44, p-value =0.009).

*SRD5A2 (TA)0-9 repeats*

The ratio of 00 and 09 TA repeats is significantly higher among the controls compared to the subjects with PCa and the difference is statistically significant (OR: 0.39, 95% CI: 0.16-0.98, p-value =0.045, and the corresponding RR: 0.84, 95% CI: 0.71-0.99, p-value = 0.046). Also, the ratio of (09+99)/00 for TA repeats is significantly higher among the subjects with PCa compared to the controls and the difference is statistically significant (OR: 2.41, 95% CI: 1.03-5.63, p-value =0.042, and the corresponding RR: 2.0, 95% CI: 1.01-3.96, p-value = 0.046).

*The genotypic profile*

The differences in the proportions of the subjects homozygous in the potential risk-bearing alleles (i.e. f of the VDR FokI locus, and SRD5A2 (TA)9 as well as heterozygosis in these two loci) and the potential protective alleles (i.e. F of the VDR FokI locus, and SRD5A2 (TA)0 repeats) are shown in Table 4. Homozygosis in the risk-bearing alleles is rare both in the control group and the PCa group. There is no statistically significant difference in the distribution of ff99 or ff and 99 homozygosity between the control and the PCa groups (Table 4). However, 66.7% of the subjects with PCa compared to 42.0% of the controls are heterozygous in these two alleles (i.e. Ff09, Ff or 09) and the difference is statistically significant (p-value = <0.004). In terms of the protective alleles, about 52.2% of the controls compared to 24.6% of the subjects with PCa are doubly homozygous (i.e. FF00) in the two protective alleles (p-value = <0.001). However, homozygosis in one protective allele (FF or 00) was more prevalent among the subjects with PCA (62.3%) compared to the controls (43.5%) and the diffidence is statistically significant (p=0.027). Overall, 95.7% of the control subjects were homozygous in at least one protective allele (i.e. had the genotype FF00, FF or 00) compared to 87.0% of the subjects with PCa (p-value= 0.070).

## Discussion

Study of cancer as a public health problem of Lebanon gained significant attention after a national cancer registry was re-established in 2005 as an institution within the Ministry of Public Health; and two reports, Cancer 2003 and Cancer 2004, were published in 2006 and 2008, respectively. In 2004, the population of Lebanon was 3,946,342 and 7,197 cases of cancer were diagnosed in that year. About 50% of the cases were men and PCa was the third-most common cancer (behind lung cancer and bladder cancer) among Lebanese men, with an age-standardized incidence rate of 27.6 cases/100,000 (Shamseddine and Musallam, 2004). The incidence rate for prostate cancer increased to 39.2 cases/100,000 by 2008, making prostate cancer the most prevalent cancer diagnosed in Lebanon, and the trend indicates further increase of the same (Shamseddine et al., 20014). The current study was a part of the pioneering prostate cancer campaign in Lebanon for molecular research on prostatic diseases. 

**Table 1:** Allelic Distribution of VDR, CYP17 and SRD5A2 Genes among the Controls (n=69) and the Subjects with PCa (n=69)

Genes	loci/groups	Observed ratio DD: Dd: dd	Expected ratio DD’: Dd’: dd’	χ^2^	p values R or NR
VDR					
	FokI/PCa	29:36:04	32.01: 29.97: 7.01	2.79	0.094
	FokI/Cont.	45:24:00	47.09: 19.83: 2.09	3.06	0.080
	BsmI/PCa	12:37:20	13.48: 34.04: 21.48	0.52	0.470
	BsmI/Cont.	11:33:25	10.96: 33.08: 24.96	0.00	1.000
	ApaI/PCa	27:36:06	29.35: 31.30: 8.35	1.55	0.213
	ApaI/Cont.	20:45:04	26.18: 32.64: 10.18	9.88	0.002*
	TaqαI/PCa	21:40:08	24.36: 33.28: 11.36	2.82	0.093
	TaqαI/Cont.	25:36:08	26.80: 32.41: 9.80	0.85	0.356
SRD5A2					
	TA 0:9/PCa	49:17:03	47.92: 19.17: 1.92	0.88	0.348
	TA 0:9/Cont.	59:08:02	57.52: 10.96: 0.52	5.02	0.025*
CYP17					
	A1:A2/PCA	19:36:14	19.84: 34.32: 14.84	0.17	0.680
	A1:A2/Cont.	12:41:16	15.31: 34.38: 19.31	2.55	0.110

*- significant at the 0.05 level of significance. DD, Dd and dd are equivalent to; for VDR gene- FokI, FF, Ff and ff; for BsaI, BB, Bb and bb; for ApaI, AA, Aa and aa; and for TaqαI, TT, Tt and tt; for SDR5A2 TA repeats- 0/0, 0/9 and 9/9; and for CYP17 gene- MspA1I, A1/A1, A1/A2 and A2/A2, respectively. DD, Dd and dd are the observed numbers, DD’, Dd’ and dd’ are the corresponding expected numbers.

**Table 2:** The Allelic Frequencies of the VDR FokI (F and f), BsmI (B and b), ApaI (A and a) and TaqαI (T and t), the CYP 17 Gene MspA1I (A1 and A2) and the SRD5A2 Gene TA Repeats (0 and 9 repeats) among the Controls and Patients with PCa

Gene (allele)	Control	PCa	OR	95% CI	p-values
VDR (F)	114 (0.83)	94 (0.68)	1.00	Ref	
VDR (f)	24 (0.17)	44 (0.32)	0.45	0.26-0.79	0.006*
VDR (B)	55 (0.40)	61 (0.44)	1	ref	
VDR (b)	83 (0.60)	77 (0.56)	1.20	0.74-1.93	0.465
VDR (A)	85 (0.62)	90 (0.65)	1	ref	
VDR (a)	53 (0.38)	48 (0.35)	1.17	0.72-1.91	0.532
VDR (T)	86 (0.62)	82 (0.59)	1	ref	
VDR (t)	52 (0.38)	56 (0.41)	0.89	0.55-1.44	0.622
CYP17 (A1)	65 (0.47)	74 (0.54)	1	ref	
CYP17 (A2)	73 (0.53)	64 (0.46)	1.30	0.81-2.08	0.279
SRD5 A2 (TA_0_)	126 (0.91)	115 (0.83)	1	ref	
SRD5 A2 (TA_9_)	12 (0.09)	23 (0.17)	0.48	0.23-1.00	0.050*

**Table 3:** The Genotypic Frequencies of the VDR FokI (FF, Ff and ff), BsmI (BB, Bb and bb), ApaI (AA, Aa and aa) and TaqαI (TT, Tt and tt), the CYP 17 Gene MspA1I (A1A1, A1A2 and A2A2) and the SRD5A2 Gene TA Repeats (00, 09 and 99 repeats) among the Controls and Patients with PCa

Locus (alleles)	Control	PCa	OR	95%CI	p-value	RR	95%CI	p-values
VDR FokI								
FF/Ff	45/24	29/36	0.43	0.21-0.86	0.017*	0.68	0.49-0.94	0.021*
FF/ff	45/0	29/40	0.07	0.01-1.39	0.081	0.88	0.77-0.99	0.046*
(FF+Ff)/ff	69/5	65/40	0.10	0.01-1.98	0.133	0.94	0.89-0.99	0.046*
(Ff+ff)/FF	24/45	40/29	2.59	1.29-5.15	0.007*	1.67	1.14-2.44	0.009*
VDR BsmI								
BB/Bb	11/33	12/37	0.97	0.38-2.49	0.955	0.98	0.49-1.99	0.955
BB/bb	11/25	12/20	1.36	0.49-3.73	0.546	1.23	0.63-2.39	0.546
(BB+Bb)/bb	44/25	49/20	1.39	0.68-2.85	0.365	1.11	0.88-1.41	0.366
(Bb+bb)/BB	58/11	57/12	0.90	0.37- 2.21	0.819	0.98	0.85-1.14	0.819
VDR ApaI								
AA/Aa	20/45	27/36	1.69	0.82-3.49	0.158	1.39	0.87-2.21	0.161
AA/aa	20/4	27/6	0.90	0.22-3.62	0.882	0.98	0.77-1.25	0.881
(AA+Aa)/aa	65/4	63/6	0.65	0.17-2.39	0.514	0.97	0.88-1.06	0.512
(Aa+aa)/AA	49/2	42/27	0.63	0.31-1.29	0.210	0.86	0.67-1.09	0.212
VDR Taq aI								
TT/Tt	25/36	21/40	0.76	0.36-1.58	0.455	0.84	0.53-1.33	0.457
TT/ tt	25/8	21/8	0.84	0.27-2.62	0.764	0.96	0.71-1.29	0.765
(TT+Tt)/tt	61/8	61/8	1.00	0.35-2.84	1.000	1.00	0.89-1.13	1.000
(Tt+tt)/TT	44/25	48/21	1.30	0.64-2.64	0.471	1.09	0.86-1.38	0.471
CYP17 MspA1I								
A1A1/ A1A2	12/41	19/36	1.80	0.77-4.22	0.174	1.53	0.83-2.83	0.179
A1A1/ A2A2	12/16	19/14	1.81	0.65-5.01	0.254	1.34	0.80-2.26	0.264
(A1A1+A1A2)/								
A2A2	53/16	55/14	1.19	0.53-2.67	0.680	1.04	0.87-1.24	0.680
(A1A2+A2A2)/								
A1A1	57/12	50/19	0.55	0.24-1.25	0.156	0.88	0.73-1.05	0.157
SRD5A2 TA repeats								
0/09	59/8	49/17	0.39	0.16-0.98	0.045*	0.84	0.71-0.99	0.046*
00/ 99	59/2	49/3	0.55	0.09-3.45	0.526	0.97	0.89-1.06	0.531
(00+09)/99	67/2	66/3	0.66	0.11-4.06	0.651	0.99	0.92-1.05	0.649
99/(00+09)	2/67	3/66	1.52	0.25-9.41	0.651	1.50	0.26-8.69	0.651
(09+99)/00	10/59	20/49	2.41	1.03-5.63	0.042*	2.00	1.01-3.96	0.046*

*- significant at the 0.05 level of significance

**Table 4:** The Difference in the Proportions of Homozygotes in Potential Risk-bearing (i.e. f and (TA)_9_) Alleles and Potential Protective (i.e. F and (TA)_0_) Alleles among the Control Subjects and the Subjects with PCa

Genotypes	Control (n=69)	PCa (n=69)	p-values
Number of homozygotes in the potential risk-bearing alleles			
ff 99	0 (0.0)	1 (0.014)	0.316
ff or 99	2 (0.029)	5 (0.072)	0.245
ff 99, ff or 99	2 (0.029)	6 (0.087)	0.145
Number of homozygotes in the potential protective alleles			
FF 00	36 (0.522)	17 (0.246)	<0.001*
FF or 00	30 (0.435)	43 (0.623)	0.027*
FF 00, FF or 00	66 (0.957)	60 (0.870)	0.070
Heterozygosity in the two alleles			
Ff 09	2 (0.029)	7 (0.101)	0.085
Ff or 09	27 (0.391)	39 (0.565)	0.041*
Ff 09, Ff or 09	29 (0.420)	46 (0.667)	0.004*

*- significant at the 0.05 level of significance

In the present study, we examined four diallelic polymorphisms of the VDR gene; of them the FokI C/T transition is located in the exon 2, the BsmI A/G transition and the ApaI G/T transversion are located in the intron between exons 8 and 9; and the TaqαI T/C transition is located in exon 9 of the VDR gene. The FokI and TaqαI polymorphisms change one codon each without altering the amino acid sequence of the VDR polypeptide. Although none of the above mutations alters the primary structure of the VDR polypeptide, some of the mutations have been found associated with varying plasma levels of the VDR ligand (Morrison et al., 1994). Our results indicate that the FokI F allele is protective, and the f allele of the same locus is associated with PCa. We found no significant association of VDR BsmI, ApaI and TaqαI alleles with PCa risk or specific protection. A study on Pakistani population indicated that the ApaI A allele is protective and the FokI and TaqαI polymorphisms have no appreciable association with PCa (Yousaf et al., 2014). Earlier, Maistro and colleagues (2004) found no association of ApaI and TaqαI polymorphisms and the risk for PCa in a Brazilian population. In another population study, Cheteri et al., (2004) found no association between the risk of PCa and FokI and BsmI polymorphisms of the VDR gene. Taylor et al., (1996) observed the tt homozygosis to be associated with a reduced risk of PCa. Another study on a Taiwanese population revealed no association of ApaI and TaqαI polymorphisms with the risk of PCa but the study found the B allele of the BsmI polymorphism as a risk factor for PCa (Huang et al., 2004), and a nearly identical result was observed previously in a Japanese population (Habuchi et al., 2000). In all, a consensus on the association of the VDR alleles with the PCa risk, severity or prognosis of PCa had not emerged; although it is apparent that the association, if there is any, is affected by the race or ethnicity (reviewed in Dianat et al., 2009).

For the CYP17 gene, we studied a single diallelic polymorphism, the MspA1I T/C transition in the 5’UTR of the exon 1 (the absence of the MspA1I site is considered the A1 allele and the presence of the restriction site is considered the A2 allele). This transition creates a binding site for the transcription factor Sp1, although it is uncertain if Sp1 actually interacts with the binding site (Nedelcheva et al., 1999). Our result indicates that the CYP17 MspA1I alleles have no association with PCa risks. Previous studies indicated that the A1 allele is associated with increased risk of PCa (Habuchi et al., 2000b, Wadelius et al., 1999). However, other groups have found an association of the A2 allele with increased risk for PCa (for example, Song et al., 2016; Stanford et al., 2002; Gsur et al., 2000). Yet another study (Sivonova et al., 2012) failed to establish any clear relationship between one or the other allele with the risk of PCa. A meta-analysis of over 25 different studies (Cai et al., 2012) indicated that it is unlikely that the MspA1I polymorphisms of the CYP17 gene affect the risk of PCa. 

Of the several polymorphisms of SRD5A2 gene reported (Peters et al., 2010), we genotyped the diallelic TA dinucleotide repeats (0 or 9 repeats) present in the 3’UTR of the gene. Our results indicate that the (TA) 0 allele is protective to PCa and the (TA) 9 allele or (TA) 0/(TA) 9 heterozygosis is possibly associated with an increased risk of PCa. The (TA) 9 allele is rare in the Lebanese population and thus our result on this locus is not statistically rigorous. Some previous studies (Rajender et al., 2009; Neslund-Dudas et al., 2007) indicated that the (TA) 9 allele is associated with increased risk of PCa. Some other studies (Kachakova et al., 2016; Salam et al., 2005) found no relationship between the longer TA repeat and prostate cancer risk. An earlier meta-analysis indicated that the TA repeat polymorphism might have a modest effect on prostate cancer susceptibility (Ntais et al., 2003).

It is evident that the outcomes of the studies involving a single polymorphism of a gene of interest or a group of polymorphisms of one gene had not provided a dependable model in predicting the risk of PCa, age of onset of the disease or prognosis of the disease after therapeutic interventions (Chen et al., 2015; Dianat et al., 2009; Ntais et al., 2003). This may be due to racial and ethnic differences (Dianat et al., 2009) and due to the presence of some risk-bearing and some protective alleles of different genes in almost every person (Kitts et al., 2014; Latil et al., 2001). It is likely that the overall genotype profile of a subject regarding all the critical genes involved in prostate physiology can be a more dependable predictor of PCa, at least for specific racial and ethnic groups. To this end, we investigated if homozygosis in the risk-bearing alleles (i.e. the f allele of the VDR FokI locus, and the (TA) 9 allele of SRD5A2 gene) or the protective alleles (i.e. the F allele of the VDR FokI locus, and the (TA) 0 allele of SRD5A2 gene) are unevenly distributed among the subjects with PCa and the controls. Our data indicates that there is no statistically significant difference in the distribution of double homozygosis (i.e. ff99) between the control and the PCa groups. However, heterozygosis in the two alleles is significantly higher among the subjects with PCa compared to the control group. Finally, a significantly higher proportion of the control subjects are doubly-homozygous (i.e. FF00) for both protective alleles compared the PCa group. The result indicates that a subject heterozygous or homozygous in the risk-bearing alleles may have a higher risk for developing PCa.

The limitations of the present study are the relatively small sample sizes and the difference in the mean age of the control and the PCa groups, given that PCa is an age-related disease. However, these limitations are difficult to overcome given that Lebanon is a small country and it has a small population of older males that were willing to participate in the screening campaign. Additionally, we investigated only six alleles of three different genes that are potentially involved in PCa. Polymorphisms in many additional genes such as androgen receptor gene (Kachakova et al., 2016; Huang et al., 2003), p53, p21 and p73 genes (Simonova et al., 2015; Mittal et al., 2011), and BCL-2 gene (Lin et al., 2016) could be associated with cancers. The overall genotype profile regarding all of the genes associated with PCa could be a more dependable indicator of PCa risk of an individual. A system biology approach had been suggested to investigate the association of gene polymorphisms and prostatic diseases (Prakash et al., 2002; Luo et al., 2001). The approach is now known as the genome-wide association studies (GWAS) of SNPs and SSLPs and disease risks (Kar et al. 2016; Patnala et al., 2013). However, since SNPs are extremely common, studies like the present one involved in identification of important SNPs relevant to disease risks of different ethnic groups will remain useful.

Benign prostate hyperplasia (BPH) is another very common Lower Urinary Tract Syndrome (LUTS) in the aging men. BPH and PCa share many signs and symptoms (Sausville and Naslund, 2010) but the signs of BPH is more overt and they become evident earlier in the disease progression. Thus, establishing BPH as the causal factor for PCa could improve early diagnosis and intervention (Orsted and Bojesen, 2013). In a previous study, we investigated the polymorphisms in the VDR gene, CYP17 gene and SRD5A2 gene in Lebanese men with confirmed BPH and observed that nearly the same set of polymorphisms of these genes are associated with PCa and BPH (data not shown). These results support and extend several previous studies showing that some common genetic polymorphisms in some of the genes are associated with BPH as well as PCa (Hamasaki et al., 2002; Habuchi et al., 2000; Habuchi et al., 2000b). Although BPH and PCa are histopathologically distinct diseases and BPH is not considered a risk factor for PCa (Chang et al., 2012; De Nunzio et al., 2011), both BPH and PCa are cytoproliferative diseases with similar hormonal and inflammatory risk factors (Miah and Catto, 2014; Orsted and Bojesen, 2013). It will be a great breakthrough if certain attributes of BPH can be established as a predictive sign of PCa.
